# Loneliness in Elderly Inpatients

**DOI:** 10.1007/s11126-022-10006-7

**Published:** 2022-11-09

**Authors:** Sandra Anna Just, Magdalena Seethaler, Rosana Sarpeah, Nathalie Waßmuth, Felix Bermpohl, Eva Janina Brandl

**Affiliations:** Department of Psychiatry and Psychotherapy, Campus Charité Mitte (Psychiatric University Hospital of Charité at St. Hedwig Hospital), Charité-Universitätsmedizin Berlin, Corporate Member of Freie Universität Berlin, Humboldt- Universität zu Berlin, Berlin Institute of Health, Große Hamburger Str. 5-11, 10115 Berlin, Germany

**Keywords:** Loneliness, Depression, Hospitalization, Elderly, Geriatric, Covid

## Abstract

**Purpose**: Loneliness among the elderly is a widespread phenomenon and is connected to various negative health outcomes. Nevertheless, loneliness among elderly inpatients, especially those with a psychiatric diagnosis, has hardly been examined. Our study assessed loneliness in elderly inpatients, identified predictors, and compared levels of loneliness between inpatients on psychiatric and somatic wards. **Methods**: *N* = 100 elderly inpatients of a somatic and psychiatric ward were included. Levels of loneliness were assessed, as were potential predictors such as depression, psychological resilience, severity of mental illness, well-being, daily functioning, and psychiatric diagnosis. Analyses of group differences and hierarchical multiple regression analysis were conducted. **Results**: 37% of all inpatients reported elevated levels of loneliness. Significant predictor variables were self-reported depressive symptoms, well-being, severity of mental illness, being single and living with a caregiver. Hierarchical multiple regression analysis revealed that the full model explained 58% of variance in loneliness. Psychiatric inpatients’ loneliness was significantly higher than loneliness in somatic inpatients. When analyzing group differences between inpatients with different main psychiatric diagnoses, highest levels were found in patients with an affective disorder, followed by those treated for organic mental disorder. Since the study took place during the COVID-19 pandemic, potential influence of different measurement points (lockdown vs. no lockdown) were analyzed: Differences in loneliness depending on the phase of the pandemic were non-significant. **Conclusion**: Elderly inpatients experience high levels of loneliness, especially those with a mental disorder. Interventions to reduce loneliness in this population should address predictors of loneliness, preferably through multiprofessional interventions.

## Introduction

Loneliness is an “unpleasant and distressing” emotion [1] which is not synonymous with social isolation – people can feel lonely even when they are objectively not alone but perceive their relationships as insufficient [2, 3]. Loneliness is a common phenomenon with an estimated prevalence of 10.5% in people aged 35 to 74 years in Germany [4]. While people of all ages can feel lonely, increased rates are found among younger adults and the oldest (> 75 years) [5, 6]. In a German sample of 1022 elderly individuals (64–94 years), loneliness was reported by around 20% of particpants [7].

Loneliness can even cause pain: an fMRI study [8] showed that brain areas associated with physical pain were also activated when participants felt socially excluded in a virtual game. It has been speculated that this social pain may have developed as an evolutionary advantage to protect people from (dangerous) isolation [3]. Several risk factors for loneliness have been identified: low educational level and income, no romantic or other relationships, poor somatic health, and poor functioning [9]. Regarding loneliness in older age, a review of 38 studies found that the same risk factors applied to this age group, but additionally identified risk factors including female gender, mental health problems, and cognitive deficits [10]. Additionally, elderly people who suffer from pain are more likely to experience loneliness [11].

Loneliness in turn has been found to contribute to overall mental and physical health including increased mortality and dementia onset [9, 11–21]. Loneliness is a reliable predictor for the development and prognosis of depression and, beyond depressive symptoms, seems to negatively affect cognitive abilities in elderly people [2, 22–25].

Early on during the COVID-19 pandemic, concerns were raised that unparalleled social restrictions would exacerbate feelings of loneliness and its sequelae [26, 27]. While studies in various countries indeed found a substantial increase in loneliness levels, not everyone was equally and continuously affected [28–31]. A recent review on loneliness among the elderly during the pandemic found an increase in loneliness [32]. However, after a few months, loneliness levels in the elderly general population fell to pre-pandemic levels again, as shown in the German Socioeconomic Panel with more than 6.000 participants [30]. Loneliness in the elderly population during the pandemic was associated with female gender, living alone, receiving care, changes in daily routine, unfamiliarity with digital media, and mental illness [33–35]. Across all ages, individuals with a psychiatric diagnosis seemed to be more severely affected by social restrictions during the pandemic than the general public [36].

Although loneliness in the general elderly population has been extensively investigated, there are hardly any studies examining loneliness in the vulnerable population of elderly hospital patients and none for elderly psychiatric inpatients. One study with geriatric inpatients in Poland reported that a majority of participants felt lonely sometimes or often – associated variables were educational level, living situation, negative life events, and level of comorbidity [37]. This lack of data is concerning as, based on the outlined research, we assume elderly psychiatric inpatients to be at a heightened risk for feelings of loneliness with detrimental effects on the course and outcome of mental illness. Apart from an increased risk for loneliness due to mental illness, admission to inpatient treatment presents a disruption of people’s routines and social behavior, thereby potentially aggravating feelings of loneliness [34]. Identifying predictors of loneliness among elderly inpatients may facilitate creating interventions tailored to the specific needs of this patient group.

Our study is the first to assess loneliness in elderly psychiatric inpatients. To control for confounding variables, we compared loneliness levels between psychiatric inpatients and a control group of somatic inpatients who were all treated on the same ward at the same time in a German hospital. Our hypothesis was that loneliness levels in psychiatric inpatients would exceed those of somatic inpatients and that significant predictor variables of loneliness across both groups, such as depression, psychological resilience, severity of mental illness, well-being, daily functioning, and psychiatric diagnosis, would correspond with findings in the existing literature. Due to the current impact on society and lifestyle, the potential influence of the COVID-19 pandemic was also taken into account.

## Methods

### Participants

One hundred inpatients in the geriatric and psychiatric department for elderly people of the Psychiatric University Hospital of Charité at St. Hedwig Hospital Berlin and of the geriatric department at St. Hedwig Hospital were included (see Table 1 for detailed sample characteristics). The mean age was 76.4 years (ranged 52–93 years). Participants showed native proficiency in German language and were able to provide written consent. The study was approved by the local ethics committee. After receiving information about the study and providing written consent, participants completed the questionnaires with assistance of a trained clinician if required. Ratings were completed by trained clinicians.

**Table 1:** Characteristics of the sample

	Psychiatric inpatients (*n* = 57)	Somatic inpatients (*n* = 43)		Statistics	*p*-value
Age (years)	72.74 (9.08) ^†^	81.26 (8.62)		*t*^a^ = -4.78	< 0.001
Sex (female)	*n* = 32	*n* = 31		*χ²* ^b^ = 2.68	0.1
Main psychiatric diagnosis				*χ²* = 64.2	< 0.001
F0x	*n* = 16	*n* = 7			
F3x	*n* = 18	*n* = 3			
F1x; F2x; F4x; F6x	*n* = 23	*n* = 2			
None	*n* = 0	*n* = 31			
Somatic comorbidity	*n* = 51				
Psychiatric medication	*n* = 53	*n* = 10		*χ²* = 50.0	< 0.001
Education years	14.0 (4.81)	13.65 (3.3)		*t* = 0.39	0.7
Financial situation				*χ²* = 10.27	0.036
Rather or very bad	*n* = 19	*n* = 7			
Neutral	*n* = 22	*n* = 10			
Rather or very good	*n* = 16	*n* = 26			
Living situation				*χ²* = 7.62	0.06
Alone	*n* = 35	*n* = 33			
With partner	*n* = 9	*n* = 8			
With other reference person(s)	*n* = 1	*n* = 1			
Assisted living/home	*n* = 12	*n* = 1			
Relationship status				*χ²* = 22.98	< 0.001
Married/permanent relationship	*n* = 12	*n* = 9			
Single	*n* = 17	*n* = 3			
Divorced	*n* = 20	*n* = 7			
Widowed	*n* = 8	*n* = 24			

^†^ Mean (SD); ^a^ t-test independent samples; ^b^ χ²-test.

### Measures

*Sociodemographic and medical information* was acquired in a questionnaire and extracted from medical records.

Loneliness was assessed with the 12-item German version [38; as seen 39] of the *UCLA Loneliness Scale* [40]. Participants indicated on a four-point Likert scale in how far the items applied to them. A sum score between 12 and 48 points was calculated for each patient. Higher scores indicated higher levels of loneliness. The German 12-item version showed sufficient internal consistency in a former study [7] which, based on a reported prevalence of loneliness of 20% in the general elderly population in former studies [41, 42] and their own sample, recommended a cut-off for loneliness at the 80th percentile, i.e. >32 points.

Depressive symptoms were assessed with the 15-item version of the German *Geriatric Depression Scale* (GDS-15) [43] where participants indicated whether they had experienced symptoms of depression in the last two weeks (yes/no). The GDS-15 has good psychometric criteria [44, 45]. Scores range from 0 (no depression) to 15 (severe depression). We applied a cut-off of ≥ 5 points for depression [46].

Psychological resilience was measured with the German *Resilience Scale RS-11.* This 11-item version of the original scale that encompasses 25 items [47] has good psychometric characteristics [48]. It is the 11-item version (Wagnild & Young, 1993). Nine items belong to the sub-scale “personal competence”, two items assess “acceptance of self and life”. Participants indicated their level of agreement on a 7-point Likert scale. A sum score (11–77 points) was calculated with higher scores representing higher resilience.

Subjective well-being was measured with the 5-item *WHO-Five Well-Being Index* [WHO-5; 49] where participants rated how often they had experienced a certain mental state in the past two weeks, from 0 (at no time) to 5 (all of the time). A sum score was generated with higher scores indicating higher well-being.

The *Clinical Global Impression – Severity Scale* (CGI) [50] allows trained clinicians to rate the severity of a patient’s illness on a scale from 1 (not at all ill) to 7 (extremely severely ill). In this study, the CGI was used to indicate the severity of mental illness, i.e. independent of severity of somatic illness.

The *Barthel Index* [BI; 51] was used following the Hamburg Manual [52] to measure the functioning of ten activities of daily living (ADL). ADL for example include eating, continence, or mobility. A sum score 0-100 is calculated with 100 points representing full functioning.

### Statistical Analysis

Statistical analysis was performed using IBM SPSS Statistics for Windows, Version 26.0 (Armonk, NY: IBM Corp.). For group comparisons, assumptions for parametric tests were examined (normality as assessed by the Shapiro-Wilk-test and visual examination regarding outliers and distribution). Depending on distribution and level of measurement of the dependent variable, the appropriate statistical test was selected. Differences between multiple groups were assessed using analysis of variance, while analyses of differences between two groups were *t*-tests or *χ*²-tests. A hierarchical multiple regression analysis was performed to identify predictor variables for loneliness across all inpatients. Before analysis, all continuous variables were z-transformed to enable comparability. Dummy variables were created for categorial predictor variables before inclusion in the regression model. Due to *n* = 16 missing values in education years, the variable was excluded from regression analysis (this should have had no effect on the results since correlation of education years with loneliness was *r* = − .03, *p* = .79). Missing values for sum scores on questionnaires led to the exclusion of *n* = 8 individual cases. All assumptions for multiple regression analysis were examined: The relationship between loneliness and independent continuous variables was checked for linearity. Data was examined for outliers (casewise diagnostics, studentized residuals, Cook’s distance, leverage) which resulted in excluding one individual observation from regression analysis. Neither auto-correlation (Durbin-Watson = 2.12) nor multicollinearity was an issue (VIF scores < 6.2). Bootstrap confidence intervals (with 1000 samples) were reported for regression weights to deal with violations of homoscedasticity and normality of residuals. Model 1 contained socio-demographic variables. In Model 2, variables describing psychopathology and functioning were added. Regression analysis was performed with *n* = 91 cases. For all analyses, the significance level was set at *p* < .05.

## Results

### Loneliness in Elderly Inpatients and its Predictors

Assessments took place between August 2020 und October 2021 with 70% of participants being interviewed after May 2021 when the second lockdown due to the COVID-19 pandemic ended in Germany. There were no significant group differences in outcome variables between participants that were interviewed during and after the lockdown.

More than one third of participants (37%) reported loneliness levels exceeding the cut-off point of 32 points, which was defined based on a prevalence of 20% of elevated loneliness in the general elderly population (Zebhauser et al., 2014).

A multiple hierarchical regression analysis was performed in two blocks to identify predictor variables for loneliness across all inpatients. An overview is presented in Table 2. Model 1 contained the socio-demographic variables age, sex, relationship status, living situation, and financial situation. The model was significant and explained 29% of variance in loneliness (*R²* = 0.37, adjusted *R²* = 0.29, *F*(10,80) = 4.74, *p* < .001).

**Table 2:** Results of hierarchical multiple regression analysis predicting UCLA-12 loneliness (*n* = 91)

		Bootstrap †		
	*b*	*SE*	95% *CI*	*p*-value
Model 1				
Constant	− 0.85	0.31	[-1.52;0.24]	0.005
Age	− 0.12	0.12	[-0.40;0.09]	0.331
Sex	− 0.02	0.23	[-0.46;0.43]	0.943
Relationship status ^a^				
Single	1.87	0.37	[1.07;2.58]	0.001
Divorced	1.00	0.29	[0.41;1.58]	0.001
Widowed	0.86	0.34	[0.27;1.65]	0.009
Living situation ^b^				
With partner	0.75	0.36	[0.003;1.48]	0.030
With caregiver/ reference person	− 0.26	0.29	[-0.90;0.34]	0.333
Assisted living	0.15	0.27	[-0.34;0.69]	0.544
Financial situation ^c^				
Neutral	− 0.53	0.21	[-0.95;-0.11]	0.014
Rather or very good	− 0.06	0.24	[-0.53;0.39]	0.800
Model 2				
Constant	− 0.49	0.45	[-1.44;0.32]	0.250
Age	− 0.05	0.10	[-0.27;0.12]	0.613
Sex	− 0.23	0.17	[-0.61;0.08]	0.162
Relationship status ^a^				
Single	1.30	0.45	[0.48;2.28]	0.004
Divorced	0.43	0.41	[-0.35;1.31]	0.255
Widowed	0.65	0.44	[-0.14;1.56]	0.113
Living situation ^b^				
With partner	0.23	0.47	[-0.59;1.23]	0.611
With caregiver/ reference person	− 0.47	0.23	[-0.90;0.04]	0.049
Assisted living	− 0.30	0.28	[-0.86;0.23]	0.243
Financial situation ^c^				
Neutral	− 0.12	.21^b^	[-0.51;0.30]	0.562
Rather or very good	0.09	0.22	[-0.31;0.51]	0.700
Depression (GDS-15)	0.56	0.12	[0.36;0.82]	0.001
Barthel Index (BI)	− 0.03	0.09	[-0.22;0.14]	0.725
Resilience (RS-11)	0.12	0.09	[-0.06;0.30]	0.201
Well-being (WHO-5)	− 0.36	0.10	[-0.57;0.15]	0.001
Severity of mental illness (CGI)	0.24	0.09	[0.06;0.41]	0.007

^†^ Bootstrap results based on 1000 samples; ^a^ reference category: relationship status: married/ serious relationship; ^b^ reference category: living situation: living alone; ^c^ reference category: living situation: living alone; UCLA-12 = UCLA Loneliness Scale (12 items); GDS-15 = Geriatric Depression Scale (15 items); BI = Barthel Index; RS-11 = Resilience Scale (11 items); WHO-5 = WHO-Five Well-Being Index; CGI = Clinical Global Impression – Severity Scale.

Significant predictors in Model 1 were relationship status (being single, divorced or widowed in contrast to being married/in a serious relationship), living situation (living with a partner in contrast to living alone), and financial situation (evaluating one’s financial situation as neutral in contrast to rather or very bad).

Model 2 additionally included variables describing psychopathology (depressive symptoms, resilience, well-being, severity of mental illness) and the Barthel-index for ADL functioning. Model 2 was also significant and explained 58% of variance in loneliness (*R²* = 0.65, adjusted *R²* = 0.58, *F*(15,75) = 9.14, *p* < .001). The change in *R²* in Model 2 as compared to Model 1 was significant (*p* < .001).

Regression coefficients for Model 2 revealed that among predictors included in Model 1 the relationship status “single” (people who defined themselves as single showed higher ratings of loneliness than married people; *b* = 1.3, 95% Bootstrap CI [0.51; 2.22], *p* = .001) remained significant. Additionally, people who lived with a caregiver or reference person other than a partner had significantly lower loneliness levels than people who lived alone (*b* = − 0.47, 95% Bootstrap CI [-0.91; − 0.03], *p* = .04). Other significant predictors in Model 2 were depressive symptoms as assessed with the GDS-15 (*b* = 0.56, 95% Bootstrap CI [0.37; 0.81], *p* = .002), severity of mental illness as assessed with the CGI (*b* = 0.24, 95% Bootstrap CI [0.06; 0.41], *p* = .009), and well-being measured with the WHO-5 (*b* = − 0.36, 95% Bootstrap CI [-0.55; − 0.17], *p* = .002).

### Psychiatric vs. Somatic Inpatients

As shown in Table 1, psychiatric inpatients were significantly younger than somatic inpatients (*p* < .001) and were more often single or divorced while somatic inpatients were more often widowed (*p* < .001). Moreover, psychiatric inpatients significantly more often rated their financial situation to be rather or very bad than somatic inpatients (*p* = .036). Unsurprisingly, psychiatric more often than somatic inpatients had a psychiatric diagnosis (*p* < .001) and took psychiatric medication (*p* < .001). Other group differences in sociodemographic or medical variables were not significant.

Psychiatric inpatients reported a significantly higher level of loneliness (*p* < .001) compared to the geriatric inpatients (on average 5 points on the UCLA-12 scale, see Table 3). Psychiatric inpatients also had significantly higher levels of depressive symptoms (*p* = .007), with both groups’ mean depression levels exceeding the GDS-15 cut-off of ≥ 5. Psychiatric patients also showed a greater severity of mental illness than somatic inpatients (*p* < .001) as well as lower levels of resilience (*p* < .001). Only ADL functioning was significantly higher in psychiatric inpatients as compared to somatic inpatients (*p* = .001). Well-being did not differ significantly between groups (*p* = .06).

**Table 3:** Group differences in outcome variables between psychiatric and somatic inpatients

	Psychiatric inpatients (*n* = 57)	Somatic inpatients (*n* = 43)	Statistics	*p*-value
Loneliness (UCLA-12)	32.11 (5.87) ^†^	27.31 (4.03)	*t*^a^ = 4.82	< 0.001
Depression (GDS-15)	7.16 (3.94)	5.17 (3.2)	*t* = 2.75	0.007
Barthel Index (BI)	76.49 (24.68)	60.23 (22.62)	*t* = 3.38	0.001
Resilience (RS-11)	48.22 (14.4)	61.9 (8.79)	*t* = 5.84	< 0.001
Well-being (WHO-5)	20.28 (7.32)	23.05 (7.01)	*t* = 1.88	0.06
Severity of mental illness (CGI)	4.88 (1.0)	2.63 (1.57)	*t* = 8.2	< 0.001

^†^ Mean (SD); ^a^ t-test independent sample; UCLA-12 = UCLA Loneliness Scale (12 items);

GDS-15 = Geriatric Depression Scale (15 items); BI = Barthel Index; RS-11 = Resilience Scale (11 items); WHO-5 = WHO-Five Well-Being Index; CGI = Clinical Global Impression – Severity Scale.

### Group Differences Depending on main Psychiatric Diagnosis

Patients were divided into three different groups depending on main psychiatric diagnosis (see Table 4): affective disorder (ICD-10 code: F3x), organic mental disorder (ICD-code: F0x) and no psychiatric diagnosis. Affective and organic mental disorder were selected as 59.65% of psychiatric inpatients fell into these categories. Among patients with an affective disorder, *n* = 15 had a diagnosis of major depression, *n* = 6 had a diagnosis of bipolar disorder. Except for one patient, all patients with affective disorder were suffering from an acute depressive episode. Organic mental disorders were almost 70% diagnoses of dementia, 30% had a diagnosis of other mental disorders (e.g. organic delusional disorder).

Loneliness levels differed significantly between the three groups (*p* < .001): loneliness was highest in people with affective disorders (52% exceeding the cut-off), followed by people with an organic mental disorder such as dementia (43% exceeding the cut-off). Lowest levels of loneliness were found in patients without a psychiatric diagnosis with only one person exceeding the cut-off for loneliness (3%). Bonferroni post-hoc analysis for multiple comparisons revealed that loneliness in patients without a psychiatric diagnosis was significantly lower than loneliness in the other two patient groups, while the difference in loneliness between patients with an affective or organic mental disorder was not significant.

This order was also found for levels of depressive symptoms (F3x > F0x > no psychiatric diagnosis; group differences were significant, *p* = .004) and resilience (no psychiatric diagnosis > F0x > F3x; group differences were significant, *p* < .001). Group differences in ADL functioning were also significant (*p* = .006), being highest in people with affective disorder, followed by people with organic mental disorder and no psychiatric diagnosis. Mental illness was rated to be most severe in people with organic mental disorder followed by those with affective disorder (group differences were significant, *p* < .001). Well-being did not differ significantly between the three groups.

**Table 4:** Group differences in outcome variables depending on main psychiatric diagnosis

Main psychiatric diagnosis	F0x (*n* = 23)	F3x (*n* = 21)	None (*n* = 31)	Statistics	*p*-value
Loneliness (UCLA-12)	30.43 (6.71) ^†^	32.19 (4.98)	26.13 (2.31)	*F*^a^ = 11.24	< 0.001
Depression (GDS-15)	6.39 (3.63)	8.37 (3.8)	4.85 (3.22)	*F* = 5.96	0.004
Barthel Index (BI)	65.65 (22.27)	78.33 (21.06)	57.58 (22.91)	*F* = 5.47	0.006
Resilience (RS-11)	55.79 (11.78)	45.41 (17.2)	61.93 (9.02)	*F* = 10.6	< 0.001
Well-being (WHO-5)	20.14 (6.74)	21.9 (7.42)	23.3 (7.4)	*F* = 1.22	0.3
Severity of mental illness (CGI)	4.87 (1.36)	4.33 (1.02)	2.32 (1.58)	*F* = 26.1	< 0.001

^†^ Mean (SD); ^a^ analysis of variance; UCLA-12 = UCLA Loneliness Scale (12 items); GDS-15 = Geriatric Depression Scale (15 items); BI = Barthel Index; RS-11 = Resilience Scale (11 items); WHO-5 = WHO-Five Well-Being Index; CGI = Clinical Global Impression – Severity Scale.

## Discussion

Our study is the first to assess subjective feelings of loneliness in elderly psychiatric inpatients in comparison with elderly somatic inpatients. Reported loneliness was high in both patient groups with overall 37% of elderly inpatients exceeding the cut-off for loneliness on the UCLA loneliness scale. As hypothesized, elderly psychiatric inpatients reported significantly higher feelings of loneliness than somatic inpatients. Highest reported levels of loneliness were indicated by patients with affective disorders such as major depression, followed by patients with an organic mental disorder such as dementia. Significant predictors for reported loneliness were depressive symptoms, subjective well-being, severity of mental illness, being single and living with a caregiver.

With over one third of participants exceeding the cut-off for loneliness, loneliness in elderly inpatients was high as compared to the elderly general population [5–7] suggesting that hospitalization may be associated with higher feelings of loneliness. A reason for high levels of loneliness among elderly inpatients could be that, at least for the duration of the hospital stay, they are taken out of their familiar environment and social networks – while arguably experiencing an exceptional, potentially frightening, life event. Following this disruption of daily routine, elderly inpatients may become more susceptible to loneliness and may have less coping strategies to address it as compared to younger people (less access to cellphones, reduced mobility, less stable social networks etc.) [34, 53]. On the other hand, it has been suggested that lonely people are already more prone to being admitted to inpatient treatment because they lack social support to deal with health-care issues adequately and early enough [54].

Our data revealed that psychiatric inpatients reported significantly higher levels of loneliness than inpatients treated for physical illness – on average, they reported loneliness levels that were almost 5 points higher than those of somatic inpatients. This finding corresponds with other studies highlighting increased loneliness in people with a mental disorder [55–57]. We also included patients living with dementia and found that they reported significantly higher levels of loneliness than patients without a psychiatric diagnosis. The prevalence of loneliness in people with dementia has been investigated in a few studies [58–60] and arguably deserves more attention. Both psychiatric and somatic inpatients reported relevant levels of depressive symptoms, on average exceeding the cut-off for depression on the GDS-15 scale. While this is not surprising for the group of psychiatric patients (more than one third of them were treated for a depressive episode), depression in the elderly without a psychiatric diagnosis may be underdiagnosed, as has been emphasized elsewhere [61–64]. Since depressive symptoms were reported in both groups, higher levels of loneliness among psychiatric patients cannot only be explained by depression. It has been suggested that stigmatization, shame, or dementia-related deficits such as aphasia may impede social interactions and thus increase loneliness [65]. Stigmatization has also been described as a cause of loneliness in other mental disorders – additional mechanisms of loneliness in mental illness include smaller social networks, social withdrawal due to psychiatric symptoms (e.g. paranoid delusions) or diminished self-worth, and reduced social or financial capital to maintain relationships [66, 67].

Hierarchical regression analysis was conducted to shed light on potential predictors for loneliness among elderly inpatients. When only including socio-demographic variables, relationship status, living situation and financial situation all explained a significant variance of loneliness. However, when adding psychopathology and functioning as predictors to the model, most socio-demographic variables were no longer significant. Only being single was associated with higher levels of loneliness – and living with a caregiver or reference person was associated with lower levels of loneliness. This is in line with former research as not having a romantic relationship and living alone have often been identified as risk factors for loneliness [9, 10, 60, 68].

Among variables of psychopathology and functioning, depressive symptoms, severity of mental illness and subjective well-being were significant predictors of loneliness across all participants. Due to the cross-sectional design of the study, no conclusions on causality can be drawn. It seems sensible to argue though that the relationship between loneliness and poor mental health is mutually enhancing. On the one hand, people who do not feel well, are depressed and show signs of more severe mental illness can experience increased levels of loneliness for reasons outlined above, namely stigmatization, social withdrawal, and lack of resources [66, 67]. Negative cognitive biases of self and others specifically can hinder people to reconnect with others and promote loneliness [69]. On the other hand, loneliness has reliably been found to enhance the risk for mental disorders, especially major depression, and to reduce well-being [2, 22, 23, 25, 57, 70].

A limitation of our study is that we did not compare loneliness with rates before the COVID-19 pandemic. Still, our findings showed no significant group differences in loneliness levels between people during a lockdown in Germany and people who did not experience restriction measures. Future research should collect longitudinal data on loneliness among hospitalized patients to allow determining potential macro-level effects on loneliness. Another limitation of our study is the cross-sectional design, not allowing for conclusions on causality.

Our study indicates potential interventions to reduce loneliness in elderly inpatients. Elderly inpatients on somatic and psychiatric wards should be screened for depressive symptoms and receive evidence-based treatment of depression if needed. Also, since loneliness seems to be especially high in elderly inpatients with a psychiatric diagnosis, psychiatric inpatient treatment for the elderly should always consider loneliness as a potentially aggravating factor in recovery. An overview of different interventions to reduce loneliness in people with mental health issues lists interventions such as changing negative cognitive biases, improving social skills, activities, and groups [71]. Groups may include “support groups, psychosocial clubs, self-help groups, mutual help groups, and trained volunteers” [67, p. 596]. A meta-analysis examining the effectiveness of interventions to reduce loneliness found that addressing negative cognitive biases in social situations was the most successful approach [71]. People with dementia, especially those in inpatient care, may be in need of specialized interventions targeting loneliness. For instance, when inpatients living with dementia do not remember visits of their loved ones, they might feel disconnected and lonely. These inpatients could benefit from specialized staff who are able to empathically communicate with them and offer comfort or connection [72]. Also, enhancing digital interactions (e.g. video calls, social media etc.) may provide a large potential to increase well-being [73] – on the condition that elderly people are enabled to increase their digital competence and use digital media appropriately [74].

## Conclusion

Our study emphasizes the importance of considering loneliness in elderly hospitalized patients, especially when they have a psychiatric diagnosis. Loneliness is most strongly associated with depressive symptoms, well-being and severity of mental illness. Also, relationship status and living situation seem to be connected to feelings of loneliness. Elderly inpatients may require interventions tailored to their needs, abilities and environment. Future studies may further evaluate the role of neuropsychiatric correlates for loneliness in elderly hospitalized patients in order to discover new strategies for diagnostic, therapy and prevention.
