# Correction to: Loneliness in Elderly Inpatients

**DOI:** 10.1007/s11126-023-10021-2

**Published:** 2023-05-03

**Authors:** Sandra Anna Just, Magdalena Seethaler, Rosana Sarpeah, Nathalie Waßmuth, Felix Bermpohl, Eva Janina Brandl

**Affiliations:** Department of Psychiatry and Psychotherapy, Campus Charité Mitte (Psychiatric University Hospital of Charité at St. Hedwig Hospital), Charité-Universitätsmedizin Berlin, Corporate Member of Freie Universität Berlin, Humboldt- Universität zu Berlin, Berlin Institute of Health, Große Hamburger Str. 5-11, 10115 Berlin, Germany

**Correction to: Psychiatric Quarterly (2022) 93:1017–1030**

10.1007/s11126-022-10006-7

In this article the author name Sandra Anna Just appeared twice. Thus, this erratum is presented to fix the error.

The original article has been corrected.

